# Battling Bias in Primary Care Encounters: Informatics Designs to Support Clinicians

**DOI:** 10.1145/3491101.3519825

**Published:** 2022-04-28

**Authors:** Lisa G. Dirks, Erin Beneteau, Janice Sabin, Wanda Pratt, Cezanne Lane, Emily Bascom, Reggie Casanova-Perez, Naba Rizvi, Nadir Weibel, Andrea L. Hartzler

**Affiliations:** University of Washington; University of Washington; University of Washington; University of Washington; University of Washington; University of Washington; University of Washington; University of California San Diego; University of California San Diego; University of Washington

**Keywords:** Implicit healthcare bias, LGBTQ+, BIPOC, clinical care communication

## Abstract

Although clinical training in implicit bias is essential for healthcare equity, major gaps remain both for effective educational strategies and for tools to help identify implicit bias. To understand the perspectives of clinicians on the design of these needed strategies and tools, we conducted 21 semi-structured interviews with primary care clinicians about their perspectives and design recommendations for tools to improve patient-centered communication and to help mitigate implicit bias. Participants generated three types of solutions to improve communication and raise awareness of implicit bias: digital nudges, guided reflection, and data-driven feedback. Given the nuance of implicit bias communication feedback, these findings illustrate innovative design directions for communication training strategies that clinicians may find acceptable. Improving communication skills through individual feedback designed by clinicians for clinicians has the potential to improve healthcare equity.

## 1 INTRODUCTION

Implicit bias–based on a patient’s race, ethnicity, gender, socioeconomic status or other prejudices and stereotypes - leads to health inequities. For example, Black patients are less likely than non-Hispanic White patients to receive appropriate treatment for heart attack[1] and pain[2, 3], and are more likely to die from childbirth[4]. Indigenous and non-White Hispanic patients have been stereotyped as being non-compliant with clinical health recommendations, having risky health behavior, and difficulty understanding and/or communicating health information[5]. In addition to these recognized inequities among Black, Indigenous, and People of Color (BIPOC), women are less likely than men to be referred for cardiovascular testing[6]. Indigenous women in the United States have high cancer disparities and have noted perceived discrimination by clinicians as a reason for not seeking recommended cancer screening[7]. Clinicians demonstrate implicit prejudices toward gay men and lesbian women[3], which exemplifies discrimination known to negatively impact healthcare quality and access for lesbian, gay, bisexual, transgender, and queer/questioning (LGBTQ+) individuals more broadly[8]. Although implicit healthcare biases may be largely unintentional, they operate at interpersonal, institutional, and societal levels. Decades of research demonstrates discrimination patients experience, driven by implicit bias, negatively impacts healthcare access, trust in clinicians, care quality, and patient outcomes[9, 10].

Mitigating implicit bias in patient-provider communication during clinical encounters is a critical opportunity for improving healthcare quality, access, and equity. Healthcare professionals exhibit the same levels of implicit bias as the wider population[11]. Clinicians’ implicit bias is not only associated with poor patient-provider communication but also with poor patient satisfaction and lack of patient trust in clinicians[12, 13]. Improvements in clinicians’ communication behaviors have the potential to disrupt negative associations between clinician implicit bias and patient satisfaction, trust, and outcomes[14]. Thus, training innovations directed at clinician communication behaviors can play a central role in mitigating implicit bias.

Although progress has been made in addressing implicit bias within healthcare, there remain major gaps in healthcare training. Despite the United States’ nationwide push to enhance education and training on implicit bias, there is room to improve existing “debiasing strategies” in healthcare[15]. Improved self-awareness of one’s own implicit bias is useful but insufficient[15] - training should also provide clinicians with concrete strategies to reduce their implicit bias[16]. Because clinicians’ implicit bias manifests in patient-provider communication, strategies that focus on improving provider communication behaviors, particularly non-verbal skills (e.g., eye contact, body language, speech tone, pitch, and volume), offer a tremendous opportunity[16]. Yet we know little about clinicians’ perspectives on opportunities for communication feedback designed to mitigate implicit bias.

The purpose of this study was to explore clinicians’ perspectives on informatics solutions to improve patient-centered communication and to mitigate clinician implicit bias. Through this research, we answer the following research question:

RQ: What design recommendations do clinicians have for feedback tools that can help improve patient-centered communication to address implicit bias in clinical interactions?

## 2 RELATED WORK

Although addressing healthcare biases is a key priority[17], there remain major gaps in measuring and mitigating the impact of implicit bias in healthcare[15, 16]. Implicit bias training has become common in medical schools and healthcare organizations through seminars and webinars[18]. While valuable, such strategies are removed from actual patient-provider interactions in which bias hides. Traditional training strategies have focused on measuring and promoting self-awareness of clinicians’ implicit bias rather than concrete tools to reduce their implicit bias, such as improving patient-centered communication[16]. Improving communication skills through individualized feedback to clinicians in the context of patient encounters is a promising direction to advance implicit bias training interventions for healthcare.

Three streams of informatics research point to opportunities to mitigate implicit bias through clinical communication training tools: traditional audit and feedback, simulated patient interactions, and automated feedback from real patient interactions. Traditional audit and feedback tools (e.g., individual performance reports) and training simulations (e.g., virtual patients), including those that focus on implicit bias, show promise. Yet this research has uncovered nuance in clinicians’ response to implicit bias communication feedback that warrants further investigation to inform the design of feedback that clinicians find acceptable. Although clinicians express interest in automated feedback on communication with real patients, prior research has not examined communication feedback associated with implicit bias. There is an opportunity to better understand clinicians’ perspectives and design ideas for tools that provide useful and acceptable feedback on biased communication behaviors with patients.

### 2.1 Traditional audit and feedback tools

Shown to improve clinician performance, confidential feedback reports (known as “audit and feedback”) refer to individual data on key quality indicators that are monitored and shared back with physicians[19]. Whether print, web-based, or embedded in electronic health records (EHRs), these reports are designed to assess care performance and lead to improvements in care quality, patient experience, and resource use. Feedback reports have potential to address implicit bias by providing clinicians with feedback on their interpersonal interactions with patients. For example, pairing feedback on implicit bias measured by the Implicit Association Test with facilitated discussion improved medical residents’ self-awareness of their implicit bias and its impact on care[20]. Yet implicit bias communication feedback can be emotionally challenging and perceived by some clinicians as threatening and lacking credibility[21], which warrants further research.

Despite being one of the most studied performance improvement interventions[22], most audit and feedback tools don’t reflect the evidence base of best practices to guide their design[19]. Scarce research investigates how to design feedback reporting systems in ways clinicians find acceptable to use and act upon. The few studies that have explored clinicians’ perceptions have uncovered nuance in the feedback process. Payne and colleagues[23] found feedback that is timely, personalized, and includes patient-level data on areas for improvement to be most acceptable to primary care providers. Yet, they also found feedback to generate resentment and negative emotion that impacted acceptability. Through design workshops with clinicians, Loomis and Montague[24] also identified nuanced reactions to feedback on patient interactions. While specific, immediate feedback was welcomed and valued by clinicians, certain types of feedback (e.g., patient surveys) were viewed with skepticism that negatively impacted perceptions of utility and trust. Given such nuance, coupled with the emotional charge of implicit bias communication feedback, understanding the perspectives of clinicians is critical to design useful and acceptable feedback reports on implicit bias in patient-provider communication.

### 2.2 Training through simulated patient interactions

A second stream of related research investigates tools that make use of simulation as a clinical communication training tool. For example, virtual patients, conversational agents, and virtual reality platforms can provide a low-pressure environment for clinician practice and training[25]. In a user study of “NERVE”, an online virtual patient simulation environment, medical students’ communication with virtual patients was rated as more empathetic than communication with human actors playing the role of standardized patients[26]. Another virtual patient platform, “MPathic-VR”, improved clinical communication training by enabling medical students to engage in challenging conversations with virtual agents[27].

Use of simulated patient interactions in implicit bias recognition and management (IBRM) curricula can be particularly valuable in fostering skill acquisition and behavior change[28]. Simulation occurs outside of real-world patient care and is thus advantageous for clinicians to confront discomfort often associated with implicit bias communication feedback. For example, simulated encounters with standardized patients can eliminate fears of actual harm to patients[29, 30]. Role-play and vignettes are also strategies to address implicit bias[31], such as videotaped social history vignettes[32]. Although some research investigates racial bias[33] and gender bias[34] with simulated patient interactions, mixed results on the effects of implicit bias training warrant further research to identify the most promising strategies[35].

### 2.3 Automated feedback on real patient interactions

A third stream of related research investigates social signal processing (SSP) systems that provide individualized feedback on clinical communication from real world patient-provider interactions. SSP is a computational approach that analyzes and synthesizes social signals observed in interpersonal interactions[36]. Social signals include nonverbal communication behaviors, such as what is said and how it is said through body movement, facial expression, and voice (e.g., talk time, interruptions, tone). Researchers have used SSP to assess clinical communication and guide feedback tools that encourage empathetic, patient-centered communication[37-40]. The rationale for SSP systems stems from the fact that verbal and nonverbal communication skills can be measured for quality[41] and improvement[42].

Two main types of SSP systems have investigated automated feedback on clinical communication: real time and reflective. Real time SSP systems are used during patient encounters. For example, “Lab-in-a-Box” provides semi-automatic segmentation of communication behaviors sensed from audio and video streams during patient encounters[40]. “Entendre” was designed to sense and convey ambient visual feedback based on nonverbal cues associated with patient-centered communication, such as talk time, turn-taking, pitch, gesture, and head nodding[38]. “ReflectLive”, which visualizes communication feedback during remote patient encounters, was found to motivate clinicians to improve their communication and increased eye contact with patients[43]. In contrast, reflective SSP systems provide communication feedback for review outside of patient encounters. For example, the “EQ Clinic” system analyzes clinicians’ communication during teleconsultations and provides graphical feedback reports which have been shown to promote self-reflection[39]. Although these automated feedback systems have demonstrated clinician interest and preliminary efficacy, none have investigated feedback on implicit bias in patient-provider communication.

## 3 METHODS

### 3.1 Procedures

We conducted qualitative semi-structured interviews with participants over Zoom. Interviews were 1-hour long and included questions about implicit biases, microaggressions, and discrimination in clinical settings. Participants also completed an online questionnaire about their clinical practice experience, demographics, self-report as BIPOC and/or LGBTQ+, experience of discrimination[44], and bias awareness[45]. The University of Washington Institutional Review Board approved study procedures.

### 3.2 Analysis

We performed thematic analysis[46] of the interview data, using Atlas.ti 10 to code the transcripts. Five researchers independently reviewed anonymized interview transcripts for codes relevant to clinician solutions for patient-centered communication and implicit bias and met as a group to discuss themes. The lead author and principal investigator then summarized the group’s analysis into thematic findings and the analysis team reviewed the summaries for relevancy. We conducted descriptive analysis of the demographic questionnaire.

### 3.3 Participants

We used snowball sampling to recruit primary care clinicians (i.e., MD, ND, NP) who provide services for people with diverse race, gender, and sexual orientation. Participants were recruited through healthcare systems in the United States. Inclusion criteria were adult (18 years or older) primary care clinicians who are fluent in English and have patients who were BIPOC or LGBTQ+ on their panel. We conducted 21 qualitative interviews (P1-P21). Participants included residents, clinic/medical directors, clinical instructors, faculty, and practicing physicians. Nearly half of the participants had 8 or more years of clinical experience. Most participants self-identified as women, White, and not Latino/Hispanic (Table 1). All but one participant (95%) reported that they had experienced discrimination in their day-to-day life based on their gender (n=11), race or ancestry (n=6), or other reason (e.g., income level, sexual orientation, age) (n=3) (Table 2).

## 4 RESULTS

### 4.1 Design recommendations

Participants shared design recommendations for feedback on patient-centered communication and implicit bias. Recommendations for types of feedback ranged from digital tools to didactic resources. Three main themes emerged in the types of digital feedback tools that clinicians recommended: (i) digital nudges, (ii) guided reflection, and (iii) data-driven feedback.

Digital nudges are recommendations for using technology tools to “nudge” a person (i.e., gently bring to one’s attention) to highlight information or behavior. Guided reflection feedback recommendations involved developing tools to help clinicians process biased communication interactions alone or with others. Data-driven feedback recommendations consisted of quantitative tools, such as dashboards for sharing automated feedback on communication cues. Participants also recommended supplementing digital tools with didactic/education tools such as diversity-focused educational resources or bias-specific clinical checklists.

Additionally, participants shared considerations for design and clinical implementation of these envisioned systems including impacts on busy clinic workflows, distractions, clinician buy-in, momentum of use over time, and privacy/confidentiality of feedback data.

### 4.2 Digital Nudges

Some participants shared an interest in using digital-based tools to “nudge” or promote patient-centered communication behaviors around implicit bias in a patient encounter. Digital nudge suggestions were recommended to be useful before, during and after a patient visit to help identify implicit biases or provide information to limit biased interaction. Before visits, smart devices, such as smartwatches, might help provide patient information to prepare clinicians with descriptive context such as a patient’s culture, pronouns, or medical needs, before visits with their patients. For example, P7 shared: “I have my smartwatch, I’m walking into the room, and it pings me and it’s like, ‘Okay. So, we’ve accessed the database and patient you’re seeing, pronoun He, She, [or They], this is their preferred name.’ This is pertinent cultural information about them. So that I can step into that room and make sure I’m providing that care”.

During visits, participants suggested apps that “helps recognize [that] what you’ve done is not necessarily an equitable thing” (P11). P1 suggested: “If feedback came through the chart as we were working on it. I mean, that probably would be the best way“. Others suggested “an alert as you open their [the patient’s] chart up and alert pops up” (P4) and “a real-time reminder of a scripted thing - in every patient encounter, you need to for two minutes turn towards that patient, not look at your computer” (P16). P14 suggested “Train people to have regular stop check-ins with their patients”. However, others expressed serious concern about such real time interventions. For example, P16 shared “the last thing I want to be doing when a patient feels uncomfortable is pay more attention to a screen or be distracted.” P18 indicated “I feel like my attention is so overtaxed…with the computer and the pieces, the clicking stuff already distracts me from the communication that if I had other flags popping up, it might be the last straw”.

After visits, a digital-based device, like a smartwatch, might help to report on clinician-patient communication issues such as “You interrupted the patient after they had been speaking for one minute” (P13). Such after-visit feedback could help the clinician recognize their communication patterns.

### 4.3 Guided Reflection

Tools that could be used to reflect on clinical interactions with patients were described by participants in several ways. We highlight three key approaches participants recommended: processing feedback with others, human observation, and practical application of communication skills.

First, participants recommended designing support for clinicians to discuss and process implicit bias communication feedback with another person or group. For example, P1 shared that time to process feedback information “in a group or just, one-on-one kind of, one-on-one regular check-ins and reflections, because you do want it to be with someone you’re really comfortable with. I think that would be hugely beneficial". Data-driven feedback alone would be insufficient for some: “And if there were automated feedback that I could also then discuss with another human being I think that would be huge” (P1). P4 recommended having a regular "health equity check in" and P1 shared “just find one other person and just do regular check-ins… just share moments where you felt your own innate biases come up”. Others suggested developing new group practices with their teams. For example, P13 suggested “having regular check-ins with our [care] team about like, ‘Let’s just do a mental exercise’.” P3 suggested to “create a culture that builds in reflection to debrief on things that happen with patients and what didn’t go so well and to make it just sort of an open culture where we talk about it and say ‘Hey, how could we have done that better?’… just creating a culture where we huddle and debrief and talk about things”.

Second, participants described the opportunity of human observation as a source of feedback, such as shadowing clinicians, where someone could observe patient interactions and share their feedback and recommendations for improving biased communication. For example, P9 suggested having “someone who is a bystander or another nurse or someone who’s in the room, an observer who says ‘Hey, what just happened there, that was not cool.’ I mean, does that ever happen real-time? Almost never. Could that happen not real-time but at a later time?”. Others suggested human observers who are already in the room and could provide feedback, such as medical assistants, cultural navigators, and other team members. For example, P4 shared “I can talk to the medical assistant later and patch things up with her, but really our priority is our patient care right here…. So, I just said [to the medical assistant], ‘Hey, I know that it was unintentional, but their pronouns are they/them”’. However, this solution could be subject to power differentials: “because we have so little moments that we’re being shadowed…. The times that we have other people in the room, it’s usually with learners who may not feel comfortable giving us that feedback” (P12). P4 recounted the role of a “Yoda” to call out communication challenges during team meetings that could be implemented as a feedback strategy: “someone else texted me … ‘Why don’t you say something?’ And I was like, ‘Oh God, I really don’t want to. I don’t want to embarrass him and whatever.’ And then they were like, ‘Well if you don’t want to, then I will.’ And I happened to be the Yoda in this particular meeting…[I]t was actually my job to notice things like to call them out”.

Third, several participants suggested creating space to not only reflect but to practice new communication skills through role playing or use of standardized patients. For example, P10 suggested a “forum to practice things. So, sometimes we practice…you were somewhere and there was a microaggression and you don’t know what to say, so let’s practice”.

### 4.4 Data-Driven Feedback

Although many participants indicated that automated, real-time feedback could be stressful or distracting to their existing clinic workflow, there were suggestions for implementing feedback into data-driven systems such as electronic health record (EHR) alerts or data dashboards.

EHR alerts could be useful to help in communicating with patients by providing easy access to contextual patient data that isn’t standardized in record systems. For example, some participants suggested that information about patients’ gender or culture could be added more explicitly to EHR interfaces. P4 shared that they would like EHR interfaces to distinguish patient pronoun preferences more easily. P17 emphasized the value of objective data: “I do like the idea of getting objective feedback on your communication in a patient visit. I do think if there was some device where afterwards it would tell you, ‘You interrupted the patient after they had been speaking for one minute.’ That would actually be interesting”.

Participants also suggested data dashboard tools that could be used outside of patient encounters to reflect on a clinician’s patient panel for communication patterns across multiple patients as opposed to individual patient interactions. Participants suggested that panel-specific reports could be useful to show inequality across their patient panels: “If those glaring statistics are in your face, you’re more likely to start thinking about why is that, and what might need to happen, … if the scorecard were to prompt like, ‘It seems like your patients of color have lower rates of preventive care’ “(P10). P18 shared “[I] probably spend [equal] time with everybody though, but who knows? Maybe I don’t. I’m trying to think what kind of medications, whether they’re hypertensives or pain medications or like A1C control, I don’t know. [A dashboard] would help you to sort of see what your gaps are, like transparency is never a bad thing.” P20 told us “you really have to look at a provider interactions en masse. So, like a hundred different interactions and then pick out some themes. I think providers might appreciate that”.

Other participants suggested automated feedback that summarizes of communication cues captured during patient encounters “like the video with after kind of feedback… But you can kind of see what your body language is giving off to the patient, and you can see how you sound” (P19). For example, P16 suggested a dashboard of key indicators, such as “number of minutes in a room, number of medications prescribed, frequency of appointments, amount of time spent typing into the EHR versus looking at the patient within the patient room? If someone could give me a dashboard, that would be awesome, because then I would know what to change.” In contrast to individual patient interactions, other participants emphasized panel views. P20 told us “I think that it would be useful instead of single interactions too. You could try to record in an entirety, like a panel of interactions, because I do think that we’re always trying to do better.” Relatedly, P10 shared: “show providers if there are disparities in their treatment of patients by race, for example…show the percentages of scores by race…. if you don’t look at race, if you just say like you have a good percentage of people getting their pap smears, then it could be easy to be happy like, "Oh great. I have a good percentage of people getting their pap smears." But if it’s broken down into like, "You have a high discrepancy between white and POC patients. Your discrepancy is higher than other providers in your field”.

### 4.5 Supplementing feedback tools with didactic resources

Some participants shared the importance of supplementing communication feedback systems with clinician education and other didactic mechanisms. Continuing education may offer opportunities for clinicians to practice communication techniques and recognize personal cues of their own biases. Examples range from tips and checklists to literature and evidence-based protocols. P12 suggested “I would spend time on [a] website, like how to talk to patients about race”. Another participant suggested an “AI bot to do a 15-30 second mindfulness exercise with” to help be calm and present before meeting with a patient (P15).

P12 recommended that having educational resources that show positive examples of communication could be beneficial: “they have videos that sort of demonstrate successful effective ways to have these conversations. And I feel like that kind of tool would be super helpful to me”. Some also saw opportunities for implementing cultural education as part of continuing education to decrease bias. P8 recommended “a course that my organization, [clinic name], would do where they’d actually teach me more about the people from Somalia, how do they view medicine and what do they understand about science and what are the things that people from Somalia might be doing at home that you don’t know about…?".

Participants also suggested that patient engagement can be a challenge and some clinicians may focus primarily on diagnostics and treatment plans. For example, P14 proposed how it would be “helpful to train people to have regular stop check-ins with their patients because I do not think that people do. And training people, as part of their encounter, empower[s] the patient and as part of their workflow stop multiple times to check in with the patient and see what they think”. Although there was strong support for implementing diversity-related educational resources, several participants had recommendations for training delivery. One participant suggested that BIPOC & LGBTQ+ academics are often tasked with delivering diversity-related training in a tokenized way. Others suggested that existing continuing education diversity educational resources could be made more engaging and worth the valuable time taken to attend. For example, P8 attended a 3-hour diversity training where “an hour and a half of it was like watching some old PBS video about race, where it was, ‘Race is a social construct, and there’s more genetic differences within people of a race than between them”’. And it just seemed silly that all these doctors were sitting around watching this, I don’t know”. They suggested that education about patients’ backgrounds and cultural identities would be more beneficial than training that reiterates generic information clinicians already theoretically learned in medical school.

### 4.6 Considerations for Design

In addition to design recommendations, participants shared several important considerations for the design of implicit bias communication feedback more generally, including impacts on (i) workflow, (ii) administrative and (iii) clinician buy-in, and (iv) privacy and confidentiality.

#### Workflow.

Although many of the clinicians were enthusiastic about receiving feedback, there were concerns about impacts of real-time implementation of those solutions on their already busy clinic workflows. P20 suggested that “Most [patient] interactions are really quick. So, I don’t know how the element of time would play in. If [feedback] is in real time that would probably take up some time during interaction, which is already so precious… But I think that if the tool was used in the moment, it would just be very stressful and I’m not exactly sure how easily it would be adopted”.

#### Administrative buy-in.

Corporate culture and systemic organization-wide buy-in were discussed extensively by participants. For example, P3 discussed a need to think more about patient representation: “I feel like as a clinic structure organization system, we’re not doing enough to really think through more carefully about all the different groups, subgroups of patients that come to our clinic and how to really represent them better so that they feel that just fundamentally that the system’s not working against them”. P21 shared that “if [feedback] was required or if it was brought to them by leadership, I feel that it would be more important because I feel like you have to get the buy-in from the people who can make it happen”. P5 provided insights for systematic organization-wide buy-in for implementing feedback: “It needs to have space to breathe to allow human interaction to happen, to have reflection happen. It often does not, and I think the tricky thing is how do you create a culture that builds in reflection to debrief on things that happen with patients and what didn’t go so well and to make it just sort of an open culture where we talk about it and say ‘Hey. How could we have done that better?”’.

#### Clinician buy-in.

Clinician readiness and buy-in was also a key consideration. For example, P4 suggested “You have to be willing to be ‘called out’. P7 shared “A lot of my colleagues haven’t quite joined this train”. P20 also suggested that “recorded behavior is always different from unrecorded behavior” which they thought would distract from patient interaction and be “artificial”. Some participants saw clinician buy-in as difficult to achieve, particularly for those who feel they do not need an intervention. For example, P21 shared “a lot of people will be like, ‘There’s nothing wrong. I’ve been a doctor for 30 years. There’s nothing wrong with how I interact with people, right?’ When actually there might be a lot wrong because you’ve been a doctor for 30 years and you think that everything you’ve been doing is fine”. P11 asked “but is it really going to change behavior? And I think what you’re going to do is take shots at people’s egos and they’re just going to feel really guilty and hate people for being called out at. And again, it might do good, but I also think you’re going to do a lot of harm”. To improve clinician buy-in, participants made several suggestions, such as “bought time” (P15) or to “set aside certain days as your feedback day” (P19). Participants shared concerns for having implicit bias metrics captured for data-driven feedback and its impacts on clinician buy-in. P21 recommended any feedback be shared with clinicians at all levels in a non-punitive way, while P7 would want to see feedback provided in a “non-judgmental” manner. Moreover, some participants thought use of a tool could lose momentum over time: “even the people who are excited about it the first couple of times, they might find a reason to not have time for it” (P15).

#### Privacy/Confidentiality.

Participants shared concerns with confidentiality of feedback, particularly the comfort of feedback being private versus public, human versus technology-based delivery of feedback, and aggregate versus individual feedback. For example, P5 explained: “I think it’s difficult for people to receive feedback… Perhaps because it’s too personal. Exception would be if somebody does something that’s egregiously wrong. Then I think it is necessary to step in but sometimes it’s better to give feedback in terms of generalizations and then allow the person to apply it to the specifics then it is to drill in on something that maybe could have been handled a little bit better but really is not that big a deal”. P4 shared insight into sharing public feedback: “I think like confronting bias or inequity, it’s challenging partly because you don’t want to call people out publicly and yet calling people out publicly makes a huge difference because then everyone hears it”.

## 5 DISCUSSION

Our findings suggest that, although clinicians express concerns about using individual feedback tools to support patient-centered communication practices for identifying implicit biases—such as impacts on busy clinic workflows, buy-in, and privacy/confidentiality of data—clinicians recommended communication feedback solutions in three main categories: digital nudges, guided reflection, and data-driven feedback. Digital nudges were recommended as a solution for reminding clinicians of patient characteristics not noted in the EHR; to provide real-time alerts to encourage patient engagement and to recognize when communication may be problematic. Research on the use of nudges as interventions in healthcare settings have predominately focused on objectives related to medication management and changes to clinical care procedures such as scheduled inpatient blood pressure checks rather than on patient-provider communication[47]. Guided reflection could be used to engage other healthcare staff in processing communication feedback through meetings, clinical consult groups, or in-clinic observations or practice settings such as the use of standardized patients. Reflective strategies of this type have been seen to increase knowledge and clinical skills and have potential to effect changes in attitudes, beliefs, and assumptions which can be useful to substantiate action at an organizational level[48-54]. Data-driven feedback, such as dashboards or adaptations to EHRs, could explicate contextual patient information (i.e., gender, sexual orientation, culture), or quantitatively indicate patterns in clinician communication (e.g., time spent typing versus talking directly to a patient) or note differences in communication by patient characteristics (e.g., race). Yet, recent studies exploring the use of electronic health records to document social determinants of health (for example: race/ethnicity, education, housing insecurity, depression) show inconsistent documentation of patient characteristics across EHR systems necessitating staff training and consistent integration into existing data workflows[55, 56].

Similar to Loomis and Montague[24], participants shared that feedback was welcomed and valued but also could be emotionally charged particularly if feedback feels threatening or punitive. Our participants suggested context-dependent individual and organizational buy-in for feedback tools along with actions towards decreasing repercussions of hierarchical structures (i.e., leadership, supervisor) should be considered in designs to limit these emotions. In addition to the design recommendations, didactic resources were recommended as an important supplement to help support patient-centered and culturally appropriate communication. Improving communication skills through individualized feedback to clinicians has the potential to enhance implicit bias training interventions for healthcare. Our findings contribute the perspective of clinicians on how this feedback can be designed in ways they find useful and acceptable.

The design recommendations and further considerations offered by clinicians are consistent with the growing work in this area. That work suggests the need to think carefully about providing feedback in ways that will be most useful to clinicians while also reflecting on institutional and clinical power dynamics factors while considering incentivized rather than punitive actions. Although feedback may help balance power dynamics between patient and clinician by increasing communication transparency, participants reported concerns over feedback being seen as potentially threatening or even discounted by their colleagues and/or healthcare leadership. Our findings are also consistent with prior work on clinicians’ mixed concerns around the “feedback paradox” in which clinicians engage with feedback they may find potentially threatening to their self-concept rather than outright rejecting it[21]. There is a need to consider how implicit bias communication feedback could still be perceived by clinicians as valid and actionable despite them possibly having negative emotional reactions to feedback that they may feel threatens their identity as respectful clinicians or be viewed as punitive.

Simple recognition of biased communication behavior on its own is less likely to show long term change[16]. Sukhera and colleagues[28] recommend educational strategies grounded in transformative learning theory that incorporate recognition and management of biases. Using this model, they suggest educational strategies with three main elements—disorienting experiences, critical reflection and dialogue, and skill acquisition and behavior change. Our findings provide additional recommendations for strategies to add to these elements. Disorienting experiences involve considering something they may not have thought about before that may be uncomfortable or challenging, such as receiving results of problematic communication behavior. Our findings suggest that digital nudges to alert clinicians of behaviors and data dashboards highlighting biased communication might be additional strategies to add to this element as these types of solutions point to problematic communication that may cause clinician discomfort. Our participants also recommended reflective feedback through shadowing or having a trusted peer or other observer talk through or share supportive, non-judgmental feedback on their communication behaviors which correspond with Sukhera et. al’s[28] element of critical reflection and dialogue. Finally, reflective feedback methods, like the use of standardized patients, could also be used to practice and acquire skills and change communication behaviors by challenging implicit bias communication patterns.

Although our study engaged clinicians to provide solutions for patient communication and implicit bias, our sample was predominantly located in metropolitan areas of the Pacific Northwest of the United States. Clinicians in other geographic and rural settings may have provided different recommendations. Our sample was largely White (64%) female (67%) and non-Hispanic or Latinx, which may not be representative of clinicians with other gender, racial, and ethnic characteristics. Despite this, we conducted interviews with 9 clinicians who describe themselves as BIPOC or LGBTQ+ (1/3 of our sample) and many participants had experience with discrimination, such as microaggressions, in their day-to-day lives that they attributed to race or gender. Engaging clinicians who may have experienced discrimination is an important viewpoint that adds depth to the growing research in this space. Although, it is also possible that clinicians who have not experienced discrimination in their day-to-day lives may require design options that vary from those who have experienced discrimination. To expand on this research, we are using the clinician design recommendations and considerations reported in this paper to develop prototypes for clinicians to provide feedback on which will be used to generate implicit bias communication feedback tools.

## 6 CONCLUSIONS AND FUTURE WORK

Participants provided design recommendations for feedback tools that improve patient-centered communication to address implicit bias in clinical interactions. Our findings show that clinicians can offer a wealth of input on how these tools can be designed in usable and acceptable ways. For the emotionally charged suggestion of implicit biases, such clinician-offered ideas provide paths for both training strategies and new tools that could ultimately reduce health inequities.

Our next steps involve integrating the findings of this work into the design of a tool to provide feedback. We are going to use participatory design with clinical experts and integrate them into our team mainly composed of Informatics and HCI researchers. We expect to engage in iterative prototype sessions that will yield a final design that responds well to our observations and the input from the domain experts. Finally, we plan to develop a working system, deploy it in control environments to evaluate its efficacy, and study the feasibility of its integration in clinic settings.

## Supplementary Material

Supplemental video

## Figures and Tables

**Table 1:** Participant characteristics

**Clinician Type**	N=21
Nurse Practitioner (NP)	1 (5%)
Naturopathic Doctor (ND)	1 (5%)
Medical Doctor (MD)	19 (90%)
**Years in Practice**	
Less than 8 years	11 (52%)
8 years or more	10 (48%)
**Age**	
Mean (sd), range	39 (10), 28-63
**Gender**	
Woman	14 (67%)
Man	5 (24%)
Non-Binary	2 (9%)
**Race**	
White	13 (62%)
American Indian/Alaska Native	2 (9%)
Asian Indian	2 (9%)
Chinese	1 (5%)
Korean	1 (5%)
White & American Indian/Alaska Native	1 (5%)
White and Other race: “Lebanese”	1 (5%)
**Ethnicity**	
Hispanic or Latino	0 (0%)
Not Hispanic or Latino	21(100%)
**Self-selected identity**	
BIPOC	5 (24%)
LGBTQ+	3 (14%)
Both BIPOC and LGBTQ+	0 (0%)
Neither BIPOC nor LGBTQ+	13 (62%)
**Patient populations on panel**	
BIPOC	17 (81%)
LGBTQ+	14 67%)
Both BIPOC and LGBTQ+	9 (43%)

**Table 2:** Participant self-report on bias and discrimination

**Experience of discrimination**	N=21
	n (%)
Never	1 (5%)
At least once	4 (19%)
2-3 times	5 (24%)
4 times or more	11 (52%)
**Bias awareness**	Mean (SD)*
Personal bias	
*1. In most situations, I am objective in my decision making.*	2.9 (1.2)
*2. Biases do not usually influence my decision making.*	4.1 (1.0)
*3. Gender identity affects the types of biases that people have against other people.*	1.5 (0.7)
Societal bias	
*4. People in today’s society tend to treat people of different social groups (e.g., race, gender, class) equally.*	4.4 (1.2)
*5. Society has reached a point where all people, regardless of background, have equal opportunities for achievement.*	5.8 (0.7)
**Biases in healthcare**	
*6. In health care, bias against others is no longer a problem in the area of hiring.*	5.6 (0.6)
*7. In health care, bias against others is no longer a problem in the area of promotion.*	5.7 (0.5)
*8. In health care, bias against others is no longer a problem in the area of leadership.*	5.8 (0.4)

* Scale: 1 “strongly agree” to 6 “strongly disagree”

## CCS CONCEPTS

• Applied computing-Life and medical sciences; • Health care information systems; • Health informatics;
